# Device Thrombogenicity Emulation: An *In Silico* Predictor of *In Vitro* and *In Vivo* Ventricular Assist Device Thrombogenicity

**DOI:** 10.1038/s41598-019-39897-6

**Published:** 2019-02-27

**Authors:** Wei Che Chiu, Phat L. Tran, Zain Khalpey, Eric Lee, Yi-Ren Woo, Marvin J. Slepian, Danny Bluestein

**Affiliations:** 1 Department of Biomedical Engineering, Stony Brook University, Stony Brook, NY USA; 2 Department of Medicine and Biomedical Engineering, University of Arizona, Tucson, AZ USA; 3 Abbott Laboratories, Pleasanton, CA USA

## Abstract

Ventricular assist devices (VAD), a mainstay of therapy for advanced and end-stage heart failure, remain plagued by device thrombogenicity. Combining advanced *in silico* and *in vitro* methods, Device Thrombogenicity Emulation (DTE) is a device design approach for enhancing VAD thromboresistance. Here we tested DTE efficacy in experimental VAD designs. DTE incorporates iterative design modifications with advanced CFD to compute the propensity of large populations of platelets to activate by flow-induced stresses (statistically representing the VAD ‘Thrombogenic Footprint’). The DTE approach was applied to a VAD (MIN_DTE_) design with a favorable thromboresistance profile and compared against a design (MAX_DTE_) that generated an intentionally poor thromboresistance profile. DTE predictions were confirmed by testing physical prototypes *in vitro* by measuring VAD thrombogenicity using the modified prothrombinase assay. Chronic *in vivo* studies in VAD implanted calves, revealed MIN_DTE_ calf surviving well with low platelet activation, whereas the MAX_DTE_ animal sustained thromboembolic strokes. DTE predictions were confirmed, correlating with *in vitro* and *in vivo* thrombogenicity, supporting utility in guiding device development, potentially reducing the need for animal studies.

## Introduction

With a steadily increasing clinical burden of heart failure (HF), coupled with a persistent heart transplant donor organ shortage worldwide, ventricular assist devices (VADs) have become the standard of care for advanced and end-stage HF patients. In recent years several VADs have been granted regulatory approval for bridge-to-transplant (BTT) and/or destination therapy (DT) indications by the Food and Drug Administration (FDA)^1,2,3^. All current approved VAD designs generate non-physiological blood flow patterns, imparting supraphysiologic shear stress to circulating platelets, ultimately activating the blood hemostatic response^4^. As a result, device recipients are prone to post-implant thromboembolic complications, mandating lifelong antithrombotic regimens^5,6^. The management of these pharmacologic-al regimens remains a major clinical challenge. Thrombotic complications are routinely reported in these FDA approved device recipients^7–14^. Unfortunately current antithrombotic therapy, recently demonstrated to have limited overall efficacy^15,16^, may in fact lead to secondary severe complications, e.g., excessive bleeding events^17–20^. Device design optimization for reducing shear-induced blood damage, and for avoidance of excessive anti-thrombotic therapy, is essential for fundamentally improving device thromboresistance and overall clinical safety and efficacy.

A device thromboresistance optimization methodology, Device Thrombogenicity Emulation (DTE), was introduced by our group^21–23^. The DTE combines *in silico* numerical simulations with *in vitro* measurements by correlating device hemodynamics with platelet activity coagulation markers – before and after iterative design modifications aimed at achieving optimized thromboresistance performance. Its efficacy was previously demonstrated in prosthetic heart valves and VADs studies^21–25^. In the MicroMed HeartAssist 5 VAD for example, following its thromboresistance optimization by DTE close to a one order of magnitude reduction in platelet activity was achieved (as compared to the predecessor design on which it was based- the DeBakey™ VAD)^22^. It additionally reduced platelet activity to a level that was far lower than that of a gold standard VAD – the Thoratec HeartMate II (HMII)^24^ (the first VAD approved by the FDA for destination therapy). The DTE optimization process also achieved platelet activity reduction that was far more effective than conventional antiplatelet drugs regimen therapy, e.g., ASA and Dipyridamole which are routinely prescribed to device recipients^16,26^. This *in silico*/*in vitro* methodology can potentially reduce the research and development (R&D) costs by developing Mechanical Circulatory Support (MCS) devices that are optimized for thromboresistance before proceeding to costly *in vivo* animal experiments, and prior to the FDA device approval regulatory process.

**Figure 1:**
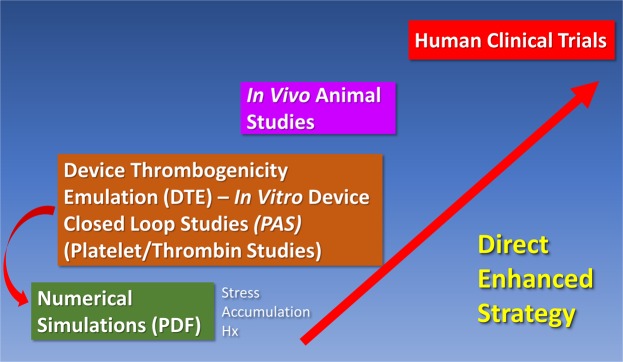
Conceptual schema for presenting the direct enhanced strategy of Device Thrombogenicity Emulation (DTE) methodology.

**Figure 2:**
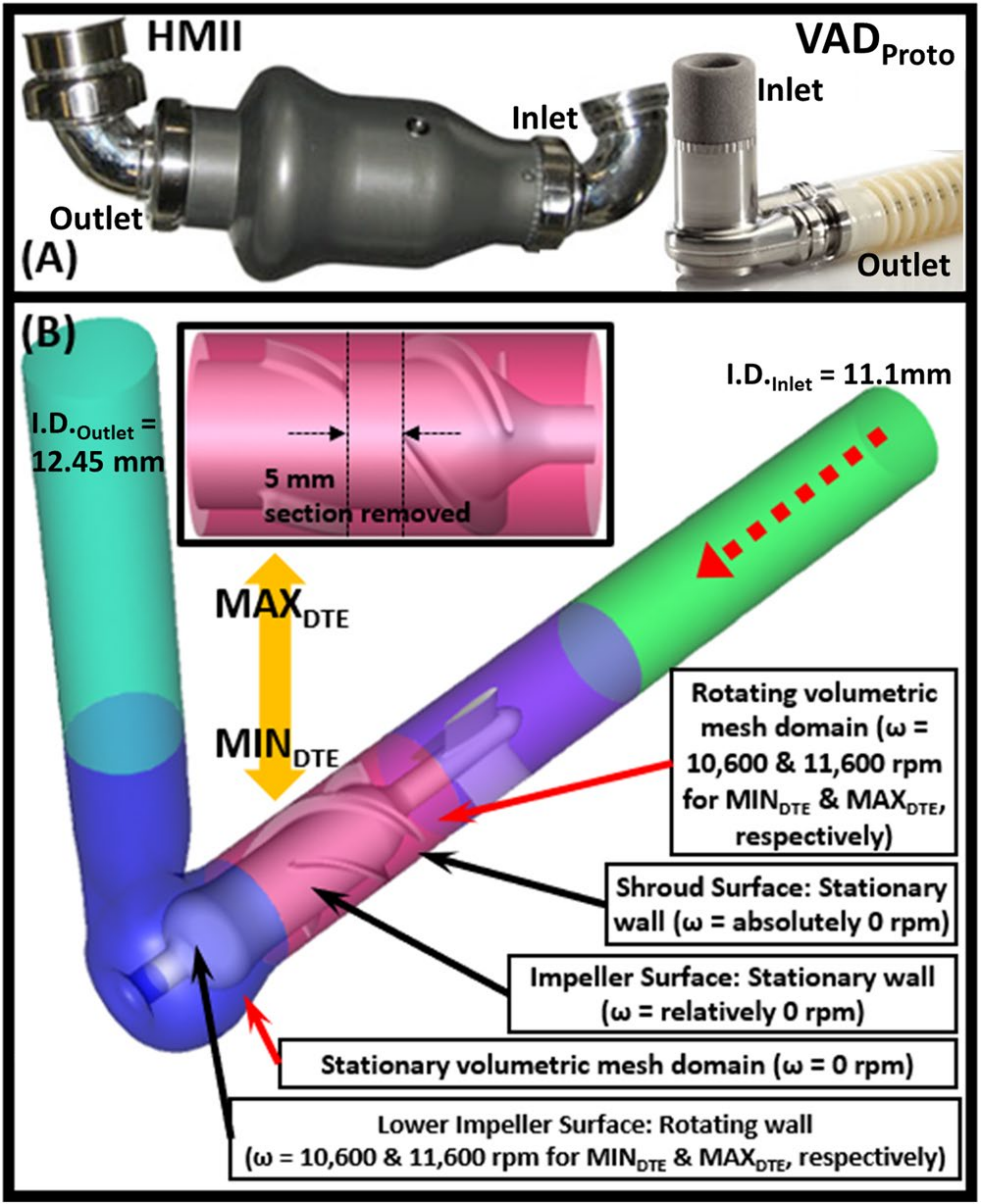
(**A**) The Thoratec HMII (left) and VAD_proto_ (right) VADs shown at the same scale. (**B**) Interior design schematics of the MIN_DTE_, and (*inset*) the modified impeller of MAX_DTE_ with a 5 mm segment of impeller blades removed. The red dotted arrow indicates the flow direction. The inlet and outlet diameters were 11.1 and 12.45 mm, respectively. The simulation boundary conditions were described in text boxes and pointed to the designated sections.

We tested the hypothesis that predictions of device thrombogenicity derived via the *in silico* DTE methodology would correlate with both *in vitro* and *in vivo* evidence of actual platelet activation and thrombosis (Fig. 1). Here a single design prototype VAD (VAD_proto_; Fig. 2A) was provided by Abbott Labs with which the authors subsequently conducted an *in silico* DTE baseline analysis. This *in silico* analysis demonstrated minimal platelet activation and represented an “optimized” baseline design (MIN_DTE_; Fig. 2B). A modification to this baseline design was simulated and fabricated to yield maximal platelet activation (MAX_DTE_; Fig. 2B, *inset*). Both prototypes underwent extensive *in vitro* comparative testing and chronic animal *in vivo* studies to evaluate and validate the differential device thrombogenicity levels predicted by the DTE methodology following the design modifications.

## Materials and Methods

### *In silico* simulations

A prototype VAD (VAD_proto_) was provided by Abbott Labs (Thoratec Corp., Pleasanton, CA – now Abbott Labs). VAD_proto_ is an axial pump, which adapts several pump design concepts from its successful predecessor – HMII (Fig. 2A). VAD_proto_ consists of a three-fin inlet stator (Fig. 2B, purple section) followed by a three blades impeller (Fig. 2B, pink section). An aft portion redirects flow outwardly 90° from the axis of the impeller (Fig. 2B, blue section). Hence, blood flow is redirected without the need for inflow and outflow elbow cannulae (Fig. 2B). Unlike the HMII VAD, and similar to current centrifugal type VAD designs, VAD_proto_ is proposed to be inserted directly into the apex of the left ventricle and is smaller and lighter than the HMII (Fig. 2A). In order to confirm the DTE approach, a VAD_proto_ pump was intentionally modified (5 mm segment of the impeller blade was removed; Fig. 2B, *inset*) with the intent to create a lower efficiency, high thrombogenicity pump (MAX_DTE_) to be used as a negative control. The comparative thrombogenic performance of the two variants was used to evaluate the efficacy of the DTE methodology.

ANSYS DesignModeler (ANSYS Inc., Lebanon, NH) was employed for geometry reconstruction in preparation for numerical mesh generation composed of solid and fluid domains that is needed for fluid structure interaction (FSI) simulations (Fig. 2B). The fluid domain of each pump was segmented into two sections – stator and rotor. For rotating mesh preparation, the fluid domain was divided into two symmetric sections according to the cylindrical symmetricity of the shroud^27^. Straight cylindrical sections with the lengths of five-times hydraulic diameters were added at the inlet and the outlet to allow for the flow to become fully developed (Fig. 2B, green and turquoise blue sections for inlet and outlet, accordingly). Interfaces were defined between these segments. ANSYS Meshing (ANSYS Inc., Lebanon, NH) was utilized for preparing tetrahedral volumetric meshes of the models. Similar mesh algorithms were applied to ensure comparable mesh densities across the devices. Extra attention was paid to interface meshing to ensure matched mesh densities across the interfaces. Rigorous mesh independence studies were conducted and were achieved with mesh densities of approx. 17 million cells for both models.

ANSYS Fluent CFD solver (ANSYS Fluent Inc., Lebanon, NH) was utilized for conducting the FSI simulations. Blood was modeled as a two-phase Newtonian fluid with viscosity and density of 0.0035 kg/m-s and 1,080 kg/m^3^. The SST k-ω turbulent model was utilized for both studies due to its superior performance for simulating transient turbulent flows and its ability to accurately predict pressure head in VAD simulations^24,28,29^. Prior to the transient simulations, steady state simulations were conducted to characterize the pumps’ performances, i.e., pre- and post- design modification, with the benchmark operating conditions – 5.3 L/min and 11.4 kPa of cardiac output and pressure head, accordingly that were provided by Abbott Labs. Moving reference frame was employed for modeling the spinning impeller – defined on the impeller segment cell zones with the designated angular velocities, with zero angular velocity applied on the shroud no-slip condition surfaces. Similarly, stationary cell zones were applied on the aft segments with designated angular velocities defined on the aft-impeller surfaces. 9.548 × 10^−2^ kg/s and 0 Pa of mass flow rate and pressure were applied as the inlet and outlet boundary conditions correspondingly for each device, and series of steady state simulations were conducted by adjusting the impeller speed until the designated pressure head of 11.4 × 10^3^ Pa was reached – 10,600 and 11,600 rpm for the MIN_DTE_ and the MAX_DTE_, correspondingly (Fig. 2B). Two-way coupled discrete phase model (DPM) was employed during the transient simulations^22,30,31^. Approx. 9.8 × 10^3^ buoyant 3 µm diameter spherical particles (998.2 kg/m^3^ of density) representing platelets were seeded and released from the upstream of both designs as flow tracers, corresponding to physiological concentration of a single cross-sectional batch of platelets traveling through the VADs. An optimized time step size of 7.53 × 10^−5^ s was utilized^24^, and a simulation duration of 0.105 s was determined for both VADs to ensure at least 90% of the platelet population had exited the flow domains^24,28^. The MIN_DTE_ and MAX_DTE_ reached 18.55 and 20.3 rotations, respectively, during the simulations.

The platelet trajectories and their instantaneous stress tensor components were recorded at each time step, and extracted subsequent to each simulation. The stress tensor of each particle was then rendered into a scalar stress value (σ)^32^.

1 $$\sigma =\sqrt{\frac{{\tau }_{11}^{2}+{\tau }_{22}^{2}+{\tau }_{33}^{2}-{\tau }_{11}{\tau }_{22}-{\tau }_{11}{\tau }_{33}-{\tau }_{22}{\tau }_{33}+3({\tau }_{13}^{2}+{\tau }_{23}^{2}+{\tau }_{13}^{2})}{3}}$$

The cumulative stresses that may drive platelets beyond their activation threshold were calculated along multiple flow trajectories as follows: the stress loading history in each platelet trajectory is calculated by summation of the instantaneous linear product of the scalar stress value and the exposure time (t_exp_) – termed Stress Accumulation (SA) according to^21,23^:

2 $$SA=\sigma \cdot {t}_{exp}={\int }_{{t}_{0}}^{{t}_{exp}}\sigma (t)dt=\sum _{i=1}^{N}{\sigma }_{i}\cdot {\rm{\Delta }}t$$

where σ_i_, *i* = *1, 2, …, N*, is the nodal scalar value extracted from the total stress tensor and Δ*t* is the corresponding time step between successive nodal points.

To statistically represent the distribution of the large ensemble of stress accumulation (SA) values− each reached by a platelet along its flow trajectory, the statistical distribution of this large SA ensemble is collapsed into a probability density function (PDFs) curve − representing the device ‘thrombogenic footprint’^22–24^ that allows for expedient comparison of the thrombogenic potential generated in the varying designs. To compare the statistical distribution of stress accumulations of different platelet populations, while guaranteeing that the percentage activation is independent of variations in the number of seeded particles and spatiotemporal variations, we have interpolated between the smaller and larger populations’ statistical distributions by applying bootstrapping statistics^23^ to guarantee that PDFs from the different population sizes are compatible and comparable.

**Figure 3:**
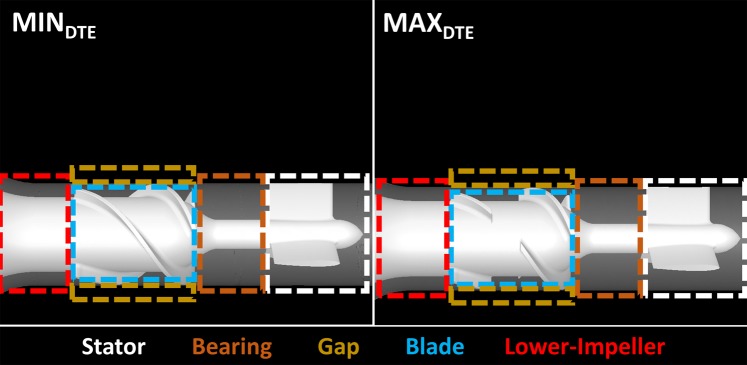
Regions of interest (ROIs) in the optimized MIN_DTE_ and the MAX_DTE_. Eight ROIs – stator, bearing, impeller-shroud gap, blade, and lower-impeller were defined for each VAD.

Regions of interest (ROI) analyses were conducted based on the cell zone segments – stator, bearing, impeller-shroud gap, blade, and lower-impeller– to evaluate the local thrombogenic potentials affected by specific design modification in these regions (Fig. 3).

### *In vitro* experiments

Abbott Labs provided two VAD_proto_ pumps for testing purposes: MIN_DTE_ and the MAX_DTE_ were prototyped by Abbott for conducting the *in vitro* and *in vivo* experiments. The operating conditions obtained through steady state simulations (i.e., 10,600 and 11,600 rpm for the MIN_DTE_ and MAX_DTE_ pump speeds, respectively, and 5.3 L/min of cardiac output) were applied for calibrating the *in vitro* recirculation flow-loop which consisted of segments of ½′′ and ¼′′ inner diameter (ID) Tygon R3603 tubes. The length of the ¼′′ ID tubes controlled the pressure head across the VADs^22,24^, and series of reducing connectors, i.e., 1/2′′ – 3/8′′ and 3/8′′ – 1/4′′ (NovoSci, Conroe, TX), were employed to avoid a sudden expansion and reduction of flow with the transition to the flow resistor^24^.

Citrated blood (120 ml) was obtained from consented healthy adult volunteers (n = 13) who had abstained from antithrombotic medications, e.g., aspirin and ibuprofen, for at least two weeks prior to blood donation. The fresh blood was collected through venipuncture according to a protocol approved by Stony Brook University IRB. Gel-filtered platelets were prepared and diluted to a concentration of 15 × 10^3^ /µl with modified Tyrode’s buffer^22,24^. For the thrombogenicity measurements the platelet-buffer solution was recirculated in the flow-loop for 30 min with 10 min sampling intervals (t = 0, 10, 20 and 30 min) utilizing the established platelet activity state (PAS) assay that reports thrombin generation rates^22,24^. PAS assay employs acetylated prothrombin to inactivate the positive feedbacks during shear-induced platelet activation to reach an one-to-one correlation between the shear dosage and the thrombin generation^33^. The linearly fitted slopes of the time sampled PAS values (normalized by thrombin generation rates achieved by platelets that are fully activated by sonication) represent the platelet activation rate (PAR) during the 30 min recirculation experiments. Experiments for both devices were conducted simultaneously using the same platelet batches to limit the study variability.

#### Statistical analysis *in silico*

The SA from the platelet trajectories was collapsed into the probability density function (PDFs) curve, which concept is an integral part of the numerical methods, and is explained above accordingly. *in vitro* – The PAR for each device was obtained by taking the linear fitting slope of the averaged time sampled PAS values. A secondary individual-experiment-based PAR calculation was achieved by averaging the linear fitting slope from individual experiments. Student’s t-test was utilized to conduct the statistical comparison of the *in vitro* results between the MIN_DTE_ and MAX_DTE_ with a significance level α = 0.05. A presumed sample size of 10, based on our previous studies, was employed before conducting sample size calculation to reach the 95% confidence level. Results are presented as the mean ± standard error of the mean (SEM), unless otherwise stated^22,24^.

### *In vivo* animal experiments

The bovine calf model has been extensively utilized to evaluate mechanical circulatory support devices^34–37^. Chronic animal studies (30 days) were conducted with the two experimental VAD prototypes implanted in young healthy adult calves (approx. 100 kg to congruent to human size) according to an IACUC protocol approved by University of Arizona (the implantation site) and Stony Brook University. The animals received humane care in compliance with the “Principles of Laboratory Animal Care” (NIH Publication No. 85-23, revised 1985). Briefly, following general anesthesia, VADs were implanted via standard left intercostal thoracotomy, the inflow cannula of the pumps were inserted into the left ventricle (LV) and secured, with outflow grafts anastomosed to the ascending aorta. Pumps were initially run at a lower speed of 7500 rpm. After verifying device functional safety, pump speed was increased to the corresponding operating rpm of each VAD prototype. Upon closing the chest, a single driveline for both operation and/or powering of the LVAD was exteriorized. Post-operatively calves were transferred from the OR and placed in sternal recumbency in an ICU stanchion cart and were monitored continuously by veterinary staff until stable, up to 7 days post-op. The veterinary staff determined when a calf could return to standard housing. The VAD implanted was battery powered by an external controller, allowing the calf to be free from the stanchion when necessary.

Routine platelet activity state measurements were conducted on blood samples extracted from the animals using the PAS assay^38,39^. Blood samples from the animals were collected in 10% ACD-A at pre-implant (before prepping for surgery), and post-operative day (POD) 0, 1, 2, 3, 4, 5, 7, 9, 14, 18, 21, 25, 28, and 30 (termination). Platelet rich plasma (PRP) was collected for the PAS assay. Chemistry panel (CP), Complete Blood Count (CBC), Basic Metabolic Panel (BMP), and Coagulation Panel were examined daily for clinical assessment. Additionally, thromboelastogram (TEG) was used to measure coagulation. The animals were kept with continuous IV heparin infusion, while bridging to warfarin (BID), maintaining ACT >220 s and INR of 1.8–2.8, respectively. 2D and M-mode echocardiography was used to analyze VAD function and cardiac flow. Euthanasia was performed according to the approved protocol at the completion of the experiment or if termination was needed for other clinical reasons. Calves were heavily sedated with Ketamine 3–4 mg/kg IV to ensure recumbency, and were given an IV barbiturate overdose (Beuthanasia-D) 1cc/10lbs IV. Post euthanasia, gross examination of devices was performed, including examination of the implant site and of device zones (intra-device, via internal examination and device sectioning). In addition, histologic evaluation of the device-tissue interface, device zones (intra-device) and detailed scanning electron microscopy of critical device internal zones and subcomponents was also performed.

#### Use of human participants

Consent was obtained for all the fresh blood donations obtained from healthy adult volunteers according to Stony Brook University Committee on Research Involving Human Subjects approved protocol (CORIHS 2012-4427-R4). All experiments were performed in accordance with relevant guidelines and regulations.

#### Use of experimental animals

Chronic animal studies were conducted according to an IACUC protocol approved by the implantation site at the Sarver Heart Center, University of Arizona (UAZ IACUC Protocol#10–193), additionally approved by Stony Brook University (IACUC 2012-1992-FAR-USDA). The animals received humane care in compliance with the “Principles of Laboratory Animal Care” (NIH Publication No. 85-23, revised 1985). All experiments were performed in accordance with relevant guidelines and regulations.

## Results

### *In silico* simulations

**Figure 4:**
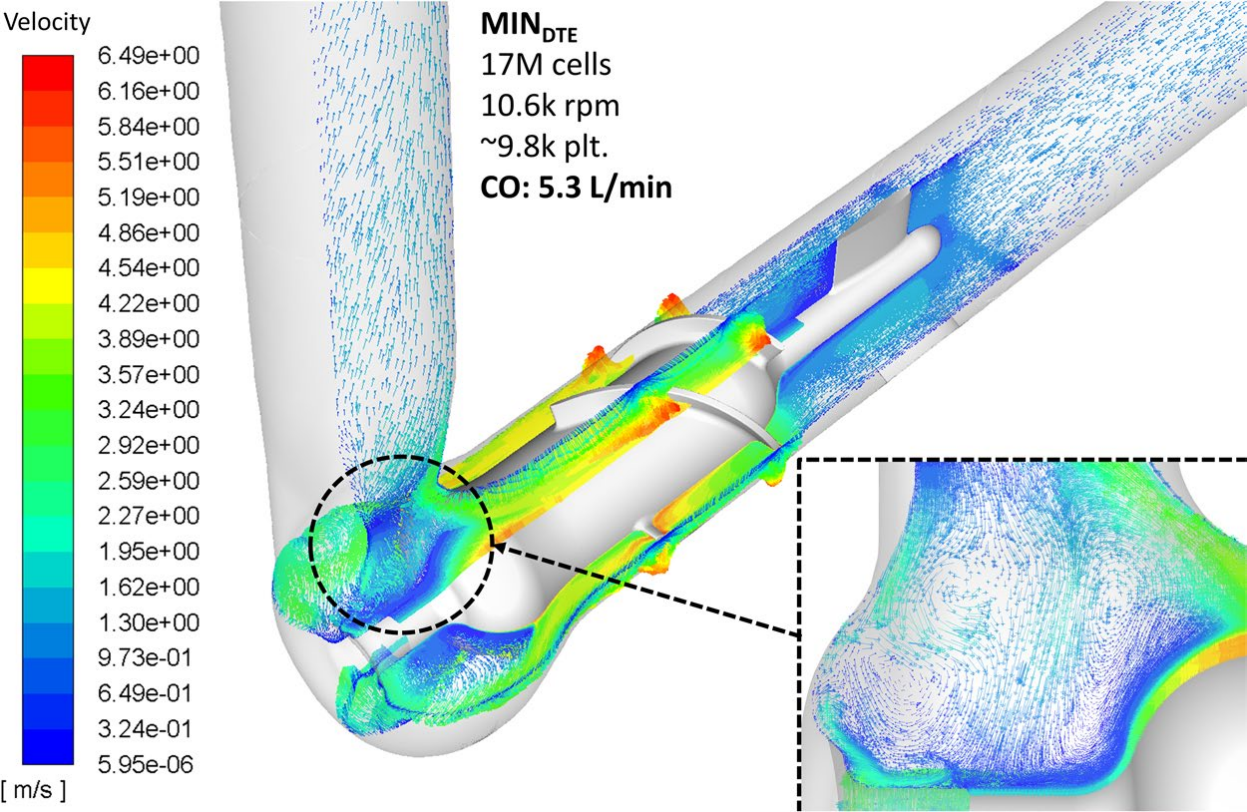
Two perpendicular cross-sections of the instantaneous velocity flow field within the MIN_DTE_ from a single freeze frame. High flow velocities were observed at the gap clearance between the impeller and the shroud. In addition, recirculation zones formed towards the outflow tract where the flow is converted from axial to centrifugal (*inset*).

**Figure 5:**
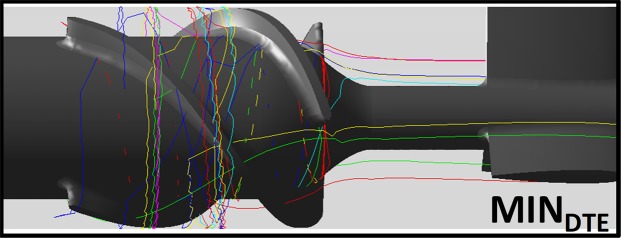
Typical platelet flow trajectories around the rotating impeller are shown (inset)- used to compute the Stress Accumulation (SA). The top ten highest SA generating platelet trajectories in the MIN_DTE_ impeller regions.

The flow is depicted by two perpendicular cross-sections showing the velocity vectors flow field within MIN_DTE_. Distinct regions of high velocities are formed at the gap clearance between the top of the impeller blade and the shroud (in red) and recirculation zones are formed towards the outflow tract where the blood flow is converted from axial to centrifugal (Fig. 4). The flow trajectories of a cluster of 9.8 × 10^3^ platelets that were released upstream (as described) was then tracked along the flow through the VAD, and used to compute for each individual platelet flow trajectory the resultant stress accumulation (SA) value that may drive the platelet beyond its activation threshold. Several highest SA generating platelet trajectories of platelets flowing through the MIN_DTE_ impeller region are shown in Fig. 5, indicating entrapped trajectory patterns along the impeller. The large SA ensemble was then collapsed into a probability density function (PDFs) curve, as explained in Methods.

**Figure 6:**
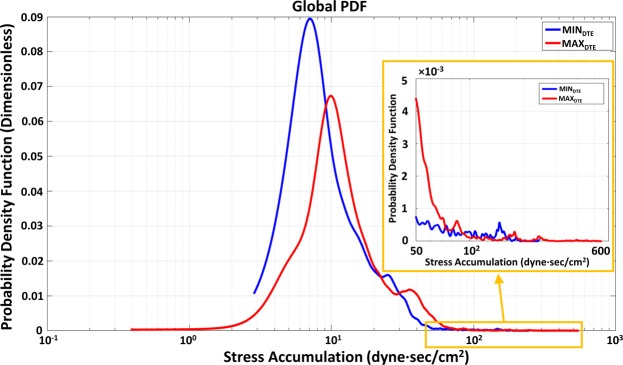
Global PDF results between the MIN_DTE_ (blue) and the MAX_DTE_ (red) VADs. The main mode of the MAX_DTE_ appeared to have wider distribution and located at the higher SA range, compared with the MIN_DTE_ main mode. Higher platelet distribution was also found in the tail region (i.e., SA >50 dyne·s/cm^2^) of the MAX_DTE_, which represented more platelets traverse through would experience higher levels of shear stress (inset).

The global PDFs of MIN_DTE_ and MAX_DTE_ (Fig. 6) indicated that: (i) the main mode of MIN_DTE_ SA distribution populated the lower SA range as compared to that of the MAX_DTE_ main mode; (ii) the MAX_DTE_ main mode had a wider SA distribution (2–30 dyne·s/cm^2^) as compared to that of the MIN_DTE_ main mode (3–15 dyne·s/cm^2^); (iii) comparing the SA distribution at the tail regions of the PDFs that are riskier, i.e., prone to activate platelets (SA >50 dyne·s/cm^2^), a secondary and tertiary modes were found for the MAX_DTE_, indicating that platelets flowing through the MAX_DTE_ have significantly higher probability to be exposed to higher SA levels than the MIN_DTE_. The secondary and tertiary modes of the MAX_DTE_ populated the highest SA range (SA >150 dyne·s/cm^2^) and extended further – as compared to that of the MIN_DTE_ secondary mode (SA range of 130–160 dyne·s/cm^2^) (Fig. 6).

**Figure 7:**
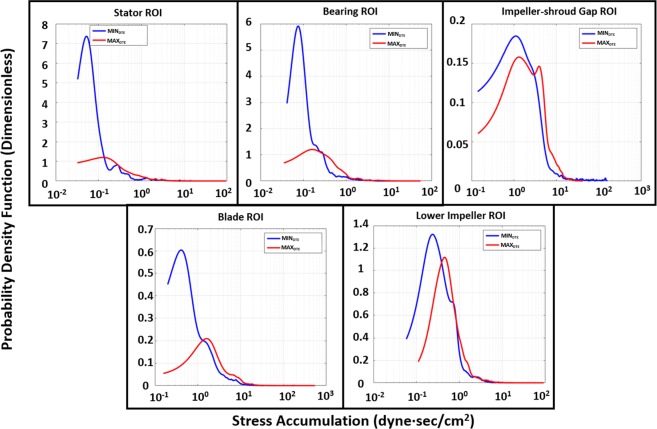
Regions of interest (ROIs) PDF results of MIN_DTE_ (blue) and MAX_DTE_ (red) VADs. The MAX_DTE_ PDFs appeared to have wider SA distribution in every ROIs comparing with the MIN_DTE_ PDFs. The MAX_DTE_ PDFs’ main modes also located at the higher SA ranges comparing to the MIN_DTE_, indicated the majority of platelets flowing through such regions in MAX_DTE_ would experience higher shear stress accumulation as compared to the platelets flowing through MIN_DTE_.

A closer look at the ROI comparative PDF results at the stator region (Fig. 7) indicates that the PDF main mode of MAX_DTE_ was shifted towards the higher SA range (to the right), with more spread out and higher probability for larger SA values distribution as compared to the MIN_DTE_. At the bearings region, the MAX_DTE_ main mode was similarly shifted towards the higher SA range, with similar SA distribution. At the impeller-shroud gap region, the MAX_DTE_ had a main bimodal distribution, and the higher larger distribution in the 4–20 dyne·s/cm^2^ SA range; however, it had a shorter higher SA range tail. Distinct differences were found at the impeller blade region, not only in the main mode of the MAX_DTE_ that populated the higher SA range and a far longer tail region distribution. For the lower impeller region, the MAX_DTE_ shared similar distribution distributions as the original MIN_DTE_; however, the entire distribution was offset toward the higher SA range (as shown in Fig. 7).

### *In vitro* platelet activity state (PAS) measurements

**Figure 8:**
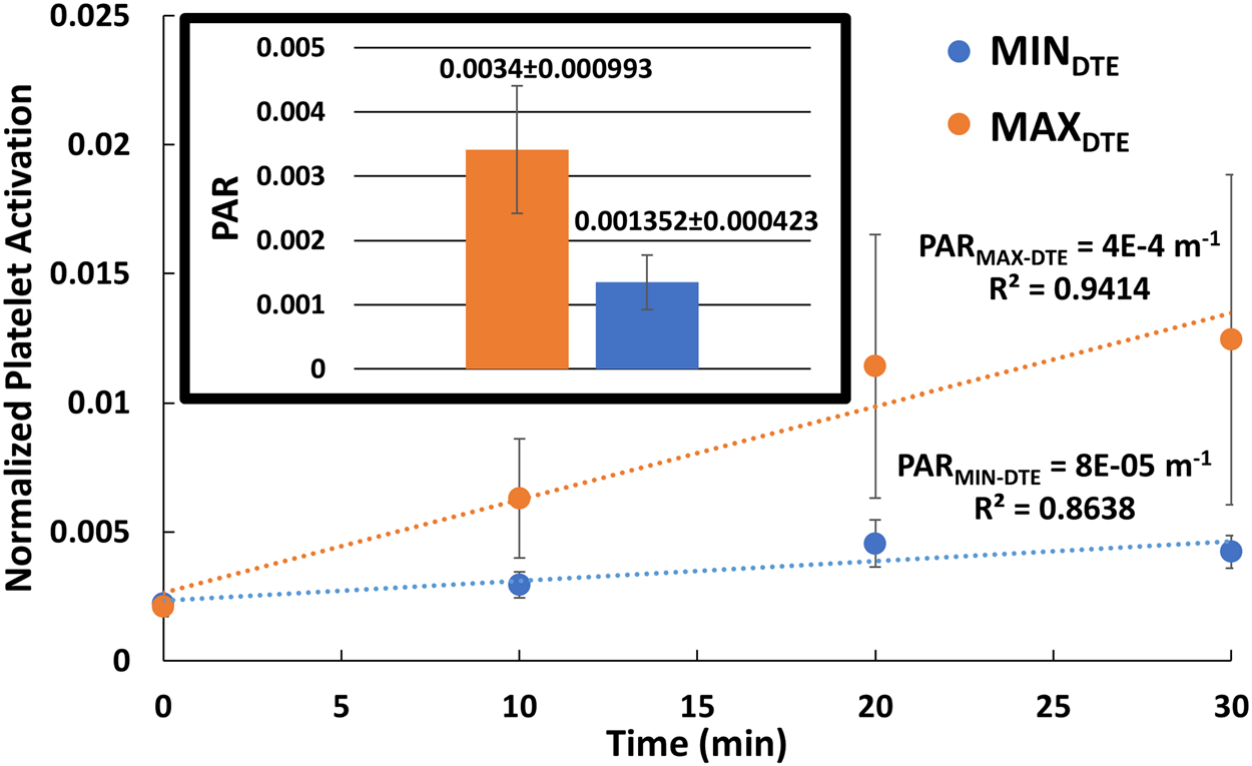
Comparison between the platelet activation rate (PAR) between the MIN_DTE_ and the MAX_DTE_ VADs. A 5-fold increase was measured in the MAX_DTE_ (*n* = *13, p* ≤ *0.03*). (*inset*) Experiment-based PAR calculation revealed a 3-fold significant increases in MAX_DTE_.

The *in vitro* measurements of the platelet activity over 30 min. recirculation time through both VADs indicated that the thrombogenicity level of the MAX_DTE_ (expressed as the platelet activity rate (PAR) ─ the slope of the PAS measurements over this circulation time) was approx. 5-fold higher than that of the MIN_DTE_ (Fig. 8; PAR = 4 × 10^−4^ m^−1^ and 8 × 10^−5^ m^−1^ for the MAX_DTE_ and the MIN_DTE_, respectively, *n* = 13, *p* ≤ *0.03*). A comparative PAR calculation based on individual experiments reveals a 3-fold differences between the MAX_DTE_ and MIN_DTE_ (Fig. 8, *inset*). This corroborated the *in silico* simulation results where the PDFs (the ‘thrombogenic footprint’) clearly showed a much higher probability of platelets being exposed to elevated SA in the case of the MAX_DTE_– strongly correlating to the measured higher platelet activation rate.

### Chronic *in vivo* animal experiments

**Figure 9:**
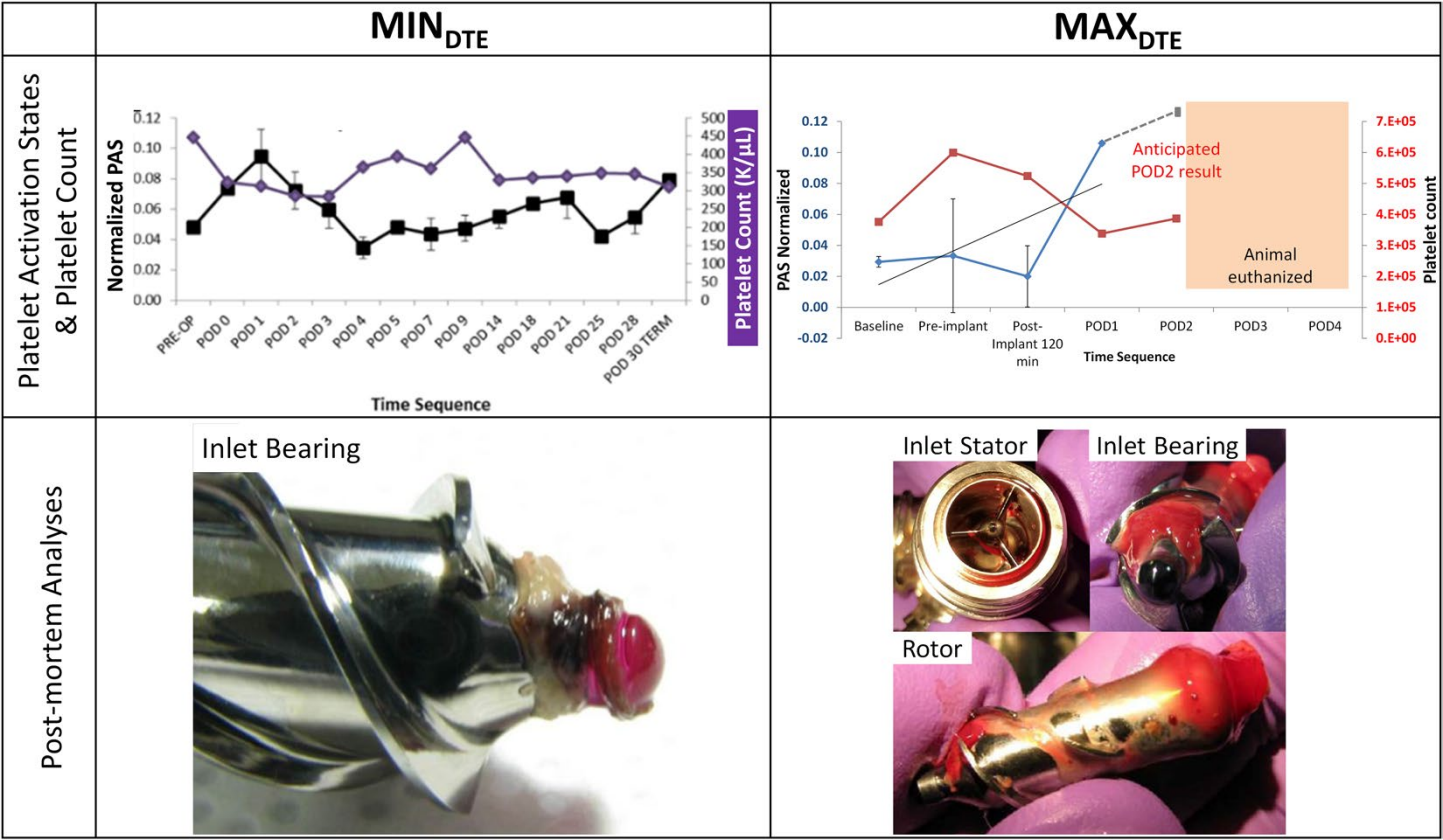
Chronic *in vivo* animal experimental results for the MIN_DTE_ (left column) and the MAX_DTE_ (right column). The top row shows the platelet activation state and platelet counts for each implanted device. The bottom row shows the post-mortem explanted devices. White thrombus was observed at the entry of the impeller in MIN_DTE_, and red thrombus was observed widely distributed along the MAX_DTE_ impeller and its aft.

Distinct differences were observed between the animals that were implanted with the MIN_DTE_ and the MAX_DTE_ in the chronic animal studies. By onset of post-implantation day 2 the platelet activity state measurements indicated that both animals experienced an acute platelet activation peak, with similar trends of platelet count found in the two animals (Fig. 9, top). The calf implanted with the MIN_DTE_ recuperated and survived well throughout the 30 days experiment. However, the calf implanted with the MAX_DTE_ did poorly, resulting in early termination (POD 2) due to a sudden seizure with accompanying incapacitating stroke from thromboembolism, following which the animal was euthanized for humane reasons (Fig. 9, right- top).

Specifically, the platelet activity state (PAS) of the calf implanted with the MIN_DTE_ peaked by POD1, likely due to the insult of surgery and peri-operative inflammation, but subsequently declined by POD4 (Fig. 9, left- top; black) with early healing. By POD5 PAS samples indicated a general trend up for the rest of the study. The activity state of platelets appeared to correlate with the white blood count and lactate dehydrogenase (LDH) levels (not shown) but not with the platelet count (Fig. 9, left- top; purple) or fibrinogen level. At the end of the 30 days chronic study, post-mortem gross and microscopic analysis revealed signs of tissue deposition on the inflow cannula and ingrowth lamination of cells on the outflow graft. There were no sign of thrombosis at the anastomosis region or the outflow graft. Enhanced fibrin deposition (hypercoagulability) was observed on the inner ostium of the inflow cannula, as well as in the disassembled VAD components− within the pump (inlet stator and inlet bearing), and on the outflow graft lining. Thrombus accumulation around the inlet bearing was examined histologically and found to consist of a homogenized, acellular eosinophilic (proteinaceous) matrix material (denatured protein) that is hypothesized to have formed as a result of heat and/or rotational force). Phosphotungstic acid-haematoxylin (PTAH) staining for fibrin confirmed the presence of condensed fibrin laminates within the thrombus material. Distinct contained regions of white thrombus were found at the inlet bearing (ball) of the impeller (Fig. 9, left- bottom).

The post mortem examination of the MAX_DTE_ explanted from the animal revealed evident thrombus in the LV adjacent to the inflow cannula and within the inflow port from the left ventricle that was 40% occluded by a red thrombus (Fig. 9, right- bottom). The outflow graft revealed thrombus in the peri-outflow region. Examination of the disassembled VAD components revealed a large red thrombus with evident adherent thrombus at the front bearing (ball) that was widely distributed from there all along the impeller, as well as around the aft region of the MAX_DTE_ impeller, likely resulting from the presence of recirculation and stagnation flow caused by the design modification (Fig. 9, right- bottom). Whitish older thrombus across the rotor was more adherent and extended from just distal to the bearing across the notch down to the aft portion of the impeller. MRI of the brain of the animal revealed large evident thromboemboli in major vessels surrounding the brain with clear evidence of parenchymal infarction.

## Discussion

The MAX_DTE_, with the partial impeller blades (5 mm gap) was expected to have a significantly higher thrombogenic potential as compared to the MIN_DTE_ design. The extensive *in silico* numerical simulations and analysis of the thrombogenic potential (via the statistical PDF distribution of the stress accumulation along a large ensembles of individual platelet flow trajectories though each device, that provides the ‘thrombogenic footprint’ of the design), clearly showed the marked differences between the two VAD designs. This was confirmed by comparing both the global PDFs of the devices, as well as by a closer examination and comparison in specific regions of interest (ROI). Physical prototype devices representing MIN_DTE_ and MAX_DTE_ were provided and tested in recirculation flow loops under typical VAD operating conditions, wherein the platelet activity generated by each was measured and compared. The *in vitro* results confirmed the numerical simulations that predicted a much higher thrombogenic potential for the MAX_DTE_. The platelet activity measurements performed *in vitro* in the actual pump prototypes operating under clinical conditions in circulation flow loops generated approx. 5-fold significantly higher thrombogenicity (in terms of platelet activity) as compared to the MIN_DTE_. This *in vitro* validation of the DTE methodology was further corroborated by the subsequent *in vivo* chronic animal studies. The animal implanted with the MIN_DTE_ prototype survived well and thrived throughout the 30 day experiments. While it was expected that the animal implanted with the MAX_DTE_ would fare inferiorly, the much higher thrombogenicity levels generated by the device led to catastrophic thromboembolic complications, leading to stroke, seizure and incapacity, with early termination of the study. The post-mortem analyses of the, e.g., MIN_DTE_, showed that white thrombus were formed at the impeller front bearing region (Fig. 9, left- bottom). This pattern closely matched the location where entrapped high SA platelet trajectories were found in the MIN_DTE_ simulations (Fig. 5), and is very similar to the thrombus formation patterns observed in explanted HMII VADs^9,10,25,40^. The red thrombus found throughout the explanted MAX_DTE_ impeller (Fig. 9, right- bottom) matched the presence of stagnation and recirculation zones caused by the intentional design modification, which additionally led to reduction of the pump efficiency. The depositions of white and red thrombus in MIN_DTE_ and MAX_DTE_, respectively, reveals the distinct flow environments of the two devices. Platelets entrapped at the entry of MIN_DTE_ impeller exposed to high shear dosage while spinning along with the higher RPM impeller, thus the high shear related white thrombus was formed^7^. The red thrombus deposited along the MAX_DTE_ impeller revealed that the fluid flow field was majorly disturbed due to the partial removal of impeller blades. Pump efficacy reduced leads to the formation of recirculation zones and stagnation flow, which leads to low-flow associated thrombus, i.e., red thrombus^7^.

## Limitations

Isolated platelets were used in the present experiments to examine the direct effect of various device associated shear environments on thrombin generation without the subsequent feedback and platelet aggregation. The platelets were diluted to a concentration of 15,000 µL due to the limited available donor blood volume and the relatively large flow loop volume. These limitations may not directly represent the physiological response of whole blood, which consists of other blood cells and plasma proteins, via which the platelet shear-induced activation is amplified. Although flow cytometry is capable of providing detailed information regarding platelet membrane glycoprotein activity, and has been utilized in several VAD studies^41,42^, here the PAS assay was utilized due to its ability to provide near-real time information on shear-induced bulk platelet thrombin generation. The DTE predictive capability would have been established more rigorously if a larger study, involving more animals, was conducted. However, such a study would necessitate near insurmountable cost and complexity. Furthermore, the present DTE experiments well demonstrate and underscore the predictive capacity of DTE. While it is recognized that DTE is far removed from the “wet” physiological scenario, such a reductionist approach is capable of predicting outcomes directly from *in silico* to *in vivo*.

*In silico* simulations and *in vitro* experiments have been widely employed during the R and D phases of VAD development. Previous VAD studies have demonstrated the robust capability of using advanced CFD to examine the intra-device flow conditions and shear environment^43,44^, leading to predictions of hemolysis^29,32^ or platelet activation^22,24^. *In vitro* experiments were mainly employed to evaluate intra-device flow environments in previous studies^45^, with some groups beginning to utilize *in vitro* experiments for examining hemolysis caused by minor design modifications^46^. However, in contrast, DTE is a pioneering concept in that it couples *in silico* simulations, *in vitro* studies and *in vivo* experiments together, developing a predictive methodology to anticipate *in vivo* trial outcomes numerically.

## Conclusions

The DTE methodology demonstrates the use of advanced numerical simulations combined with experimental techniques for quantifying measures that are directly relevant to thrombosis and coagulation markers in devices, that is predictive of how devices may perform *in vivo*. The veracity of the DTE *in silico* simulations predictive capability was validated by *in vitro* recirculation experiments, and further translated and confirmed by *in vivo* VAD implantation animal studies. The robust capability of this predictive technology demonstrates its utility as a cost-effective pre-clinical MCS thrombo-optimization approach. DTE offers the potential to transform the conventional approach currently utilized for designing and developing MCS devices. Presently numerous, costly, burdensome *in vivo* animal studies are required during each development cycle. DTE-enhanced design offers the potential of a more direct approach, with *a priori* enhanced hemocompatibility (Fig. 1), ultimately reducing both the time and cost of device development. Further, this approach may eventually obviate the need for animal experiments, providing a useful adjunct to enhance regulatory science approaches and approval.

## Data Availability

The data that support the findings of this study are available from the corresponding author upon reasonable request. The data of design iterations that were performed during this study are available from Abbott Labs, but restrictions apply to the availability of these data which are not publicly available.
